# Efficacy Analysis of a Script-based Guide for EVAR Execution: is it Possible to Reduce Patient Exposure to Contrast, Operative Time and Blood Loss even when Advanced Technologies are not Available?

**DOI:** 10.5935/1678-9741.20150079

**Published:** 2015

**Authors:** Giovani José Dal Poggetto Molinari, Ana Terezinha Guillaumon, Andréia Marques de Oliveira Dalbem

**Affiliations:** 1 Universidade Estadual de Campinas (Unicamp), Campinas, SP, Brazil

**Keywords:** Endovascular Procedures, Aortic Aneurysm, Abdominal, Multidetector Computed Tomography, User-Computer Interface

## Abstract

**INTRODUCTION:**

Despite the patient and medical staff exposure to radiation in endovascular aneurysm repair, the benefits of this abdominal aortic aneurysm type of surgical management are justfied by minor recovery time and hospitalization, as well as an option for patients not elected to conventional open repair. In this minimally invasive surgical aproach, time of procedure and radiation doses can be substantial - and the increasing frequency of these procedures and it's complexity have impelled vascular surgeons to face additional and successive risk to occupational radiation exposure. Meticulous study of the computed tomography angiography during the endovascular aneurysm repair preparation allows reduction of unnecessary radiation exposure, as also reduces consecutive image acquisition and contrast use (that may be related to renal overload in susceptible patients). Some studies have proposed strategies to optimize endovascular intervention to reduce contrast use and X-ray exposure. Although they might prove to be effective, they rely on use of additional specific and advanced equipment, available only in major centers. As an alternative to this expensive and restrict technology, it is presented a simpler technique through image manipulation on software OsiriX, aiming to reduce both exposures.

**OBJECTIVE:**

To analyze the efficacy of the adoption of a study protocol and a script-based guide in preparation for endovascular aneurysm repair through verifying it's impact over the surgical procedure - as referred to intravascular contrast infuse, effects over renal function, blood loss and operatory time.

**METHODS:**

A longitudinal prospective study from March 2014 through March 2015, where 30 performed endovascular aneurysm repair were compared to a historic control group. The planning for endovascular aneurysm repair through the patient's tomographic image manipulation in the prospective group was performed with OsiriX MD software. A script-based guide upon gathering detailed computed tomography angiography images was elaborated by the author and distributed to the performing surgical team for appreciation, instruction and pre operatory judgment. Based upon the script, the C-arm gantry angle was specifically corrected in each case of endovascular aneurysm repair, for image optimization and aneurysm's neck visualization. Arteriography was performed under digital subtraction angiography after catheters were positioned according to predicted level description in the referred guide. Statistical analysis were performed with a significance level of 5% (*P* value<0.05).

**RESULTS:**

There was a statistically significant relationship between the two studied periods and the variables: contrast volume (284.5 *vs.* 31.8 mL), operative time (207.5 *vs.* 140.4 min.) and blood loss (798.1 *vs*. 204.4 mL), revealing that they are considerably larger in the historical control group than in the script guided current group. There was no difference related to the volume of contrast used in the two groups and the occurrence of renal impairment.

**CONCLUSION:**

In the present paper it was possible to demonstrate the impact of the ability to manipulate digital formats of medical images without the need of sophisticated equipment, in adoption of a guide based on the compilation of informations collected with assistance of an accessible software performed on a personal computer. Although we could not prove relation to occurrence of renal impairment, there were direct results on reduction of intravascular contrast use, even as surgical time and blood loss, compared to a previous historical period.

**Table:** 

**Abbreviations, acronyms & symbols**	
AAA	= Abdominal aortic aneurysm
ALARA	= As low as reasonably achievable
CIN	= Contrast induced nephropathy
CMSC	= Contrast Media Safety Committee
CTA	= Computed tomography angiography
DREAM	= Dutch Randomized Endovascular Aneurysm Management
DSA	= Digital subtraction angiography
EVAR	= Endovascular aneurysm repair
FPS	= Frames per second
GFR	= Glomerular filtration rate
ICU	= Intensive care unit
NAC	= N-acetylcysteine
OVER	= Open Versus Endovascular Repair Veterans Affairs Cooperative Study Group

## INTRODUCTION

Due to its high accuracy, the computed tomography angiography (CTA) is the diagnostic modality and tool for aortic disease evaluation most widely used by vascular surgeons. Multidetector devices can acquire thinner slices in reduced time, generating high definition images resolution over one single contrast injection. Therefore, they are essential for three-dimensional reconstructions, with plenty of detailment in any selected incidence. Despite the ionizing radiation exposure, this modality of image acquisition is viable, fast, efficient and high cost-benefit^[1]^. However, the angiotomography study produces a large series of digital data with difficult handling, wherein interpretation and discrimination of the variations are better accessed when post processed in softwares, as in multiplanar reconstructions, volume rendering and maximum intensity projection^[2]^.

The evaluation of these tomographic images consists of three main tasks: determining the eligibility for endovascular aneurysm repair (EVAR), choosing the appropriate graft and simulate an intervention plan. For preoperative planning, several anatomical factors must be accessed, including neck localization and morphology in relation to major branches of the aortic axis, to establish the endograft landing zones, it's diameters to choose the device's size, the iliac and femoral arteries' conditions towards the vascular access site^[1]^.

It is known that in this endovascular modality, time of procedure and radiation doses to the patient and surgical staff may be substantial^[3]^. The increasing frequency of these procedures and its complexity has impelled vascular surgeons to face additional and successive risk to occupational radiation exposure^[4]^. Meticulous study of the CTA during the procedure's planning allows reduction of unnecessary exposure, beyond the use of consecutive contrast image acquisition - that may contribute to renal overload and chronic renal disease development, especially in patients with diabetic nephropathy or any kind of pre-existent renal impairment^[5]^.

Of these, the contrast induced nephropathy (CIN) may vary from 12% to 50% after exposure, thus it should be one of the main concerns about EVAR^[6]^. The Contrast Media Safety Committee (CMSC) states CIN as a condition whereas renal injury (an increase of 25% over serum creatinine level or 44 umol/L) occurs within 3 days after intravascular contrast infusion and no other etiology involved^[7]^.

Several studies have proposed strategies to optimize endovascular intervention in reducing renal contrast overload and ionizing radiation exposure^[3,5,8,9]^. Such steps might prove effective, however rely solely on use of additional features, specific and sophisticated equipment, available only in major centers.

Aiming to reduce both exposures related to the use of contrast and ionizing radiation, we believe instead that it's possible to use a simpler technique through image manipulation on software (OsiriX, Pixmeo Labs, Geneva, Switzerland) as an alternative to this expensive technology. In this present article we propose the adoption of a study protocol in preparation for EVAR, gathering detailed information of the aneurysm's morphologic aspects, its spatial relation to bone structures that may be visualized under fluoroscopy with immediate applicability - allowing reducing operatory time, prolonged exposure to radiation and iodinated intravascular use.

The primary aim of this study was to assess the efficacy of the adoption of a study protocol and a script-based guide in preparation for EVAR. Within the protocol application, detailed individualized data of patient's radiographic characteristics was collected and applied as a script to intraoperative steps - which allowed to verify the protocol impact over the surgical procedure, as referred to intravascular contrast infuse, effects over renal function, blood loss and operatory time.

## METHODS

A longitudinal prospective study was performed from March 2014 through March 2015. A non randomized convenience group was selected, with patients diagnosed with infrarenal abdominal aortic aneurysm (AAA) treated at the High Complexity Center for Endovascular Surgery at the State University of Campinas (Unicamp). Within this period, 30 of the 34 performed EVAR were included for statistical analysis. These surgeries were followed by the author in each step of the planning, endovascular execution and postoperative time at the intensive care unit (ICU). Patients with ruptured AAA treated by EVAR and surgeries that the author could not follow, for any reason, any part of planning or execution were excluded from statistical analysis.

All EVAR were performed over a radio-transparent table in an operatory room, equipped with a C-arm fluoroscopy device (General Electric 9800 Plus - GE OEC Medical Systems, Inc., USA), with a 15 inches visual field.

The control group (historic) is composed by 28 elective EVAR performed between January 2009 and December 2010. At that time, the preparatory plan was not made as in this present model - the main tool of this study's analysis.

For both groups, the X-ray tube was positioned under the operating table and the fluoroscopic image capture was performed in pulsed mode at a 7.5 frames per second (FPS). The digital subtraction angiography (DSA) was used at moments of image capture for aneurysm's neck visualization and control arteriography for aneurysm sac exclusion under a 15 FPS rate. Data of fluoroscopy time, DSA time, total air kerma X-ray cumulative dose were registered in the prospective group, provided automatically by the device's software. It was not possible to retrieve the same informations in the historic group, because they were not described in patients' files.

**Fig. 1:**
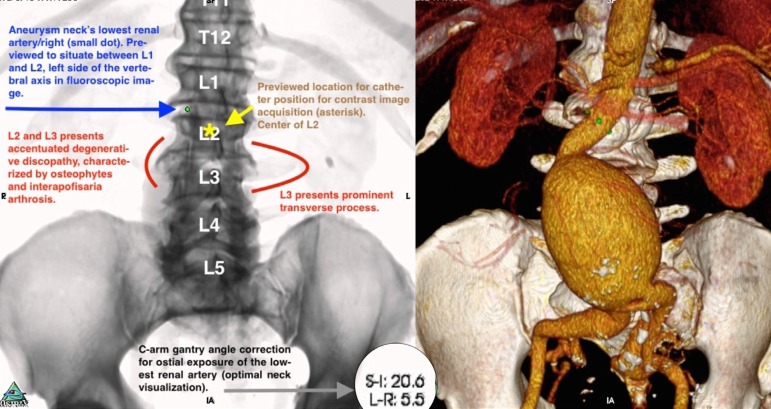
Example of a scriptbased guide. Elaboratedafter detailed study of the CTA, with preview of structures positioning under fluoroscopic view and the C-arm gantry angle correction.

The planning for EVAR through the patient's tomographic image manipulation in the prospective group was performed through OsiriX MD software (Pixmeo Labs., Geneve, Switzerland). The stages were composed of: 1) identification of the C-arm gantry angle for the ostial visualization of the neck's lowest renal artery within the virtual geometric correction^[10]^; 2) renal arteries' spatial marks and three-dimensional by volume rendering under virtual fluoroscopy preset^[11]^; 3) recording of complementary information that were essential to visualize and recognize the aneurism's neck during the intra-operatory time, such as renal artery positioning under fluoroscopic view related to bone structures (vertebrae, osteo-degenerative individual characteristics) and other details that helped to foresee angiographic catheter positioning for optimal image acquisition. A script based upon gathering these images and registered information was elaborated by the author and distributed to the performing surgical team (fellows and medical assistants) a day before the surgery for appreciation, instruction and pre operatory judgment, as exemplified (Figure 1).

Based upon the script, the C-arm gantry angle was specifically corrected in each case of EVAR, for image optimization and aneurysm's neck visualization and lowest renal artery's ostial exposure. Arteriography was performed under DSA, after insertion of the endoprosthesis infrarenal main body, with the image capture at the centre of the radioscopic field. Manual intra-arterial injection of nonionic iso osmolar iodinated contrast (Omnipaque 300 mg/mL) was performed with the patient under temporary apnea. At this moment, a guide-catheter or the prosthesis sheath itself was positioned right under the bone reference for the lowest renal artery's position (under fluoroscopic view, predicted by the virtual fluoroscopy). Endovascular devices compounded by a separated sheath (Endologix AFX and Gore Excluder), the contrast injection was made with 10 mL of pure iodinated flushed by 20 mL of saline bolus. In those conjunct to the delivery sheath (Cook Zenith, Medtronic Endurant, Lombard Medical Aorfix), the iodinated contrast was diluted to saline in equal parts of 10 mL and injected through a RDC or MP guide-catheter.

All participants were hospitalized under preoperative hydration (saline infusion of 1-1.5 ml/kg.h), administered to N-acetylcysteine (NAC) (600 mg twice daily) and had suspension of nephrotoxic drugs. Additionally, according to our institutional protocols, for patients with renal impairment (serum creatinine over 1.8 mg/dL), it was given isotonic sodium bicarbonate (150 mL Bic 8.4% diluted to 850 mL NaCl 0.9%) in a dose of 3 mL/kg of body weight 1h prior to procedure and 1 mL/kg.hr within the next 6 hours. All patients were referred to postoperative medical care at the ICU. Laboratory tests (hemogram, hydroelectrolytic and renal profile) were collected at admission and daily for the first 72h.

Statistical analysis was performed, with the Chi-square and Fisher's exact tests for two qualitative categories or likelihood ratio (more than two categories). A statistical significance level of 5% was considered (*P* value < 0.05).

## RESULTS

Comparing both groups (prospective and historical control), there was no statistically significant difference in demographic and morbid characteristics of the study treated patients.

**Fig. 2:**
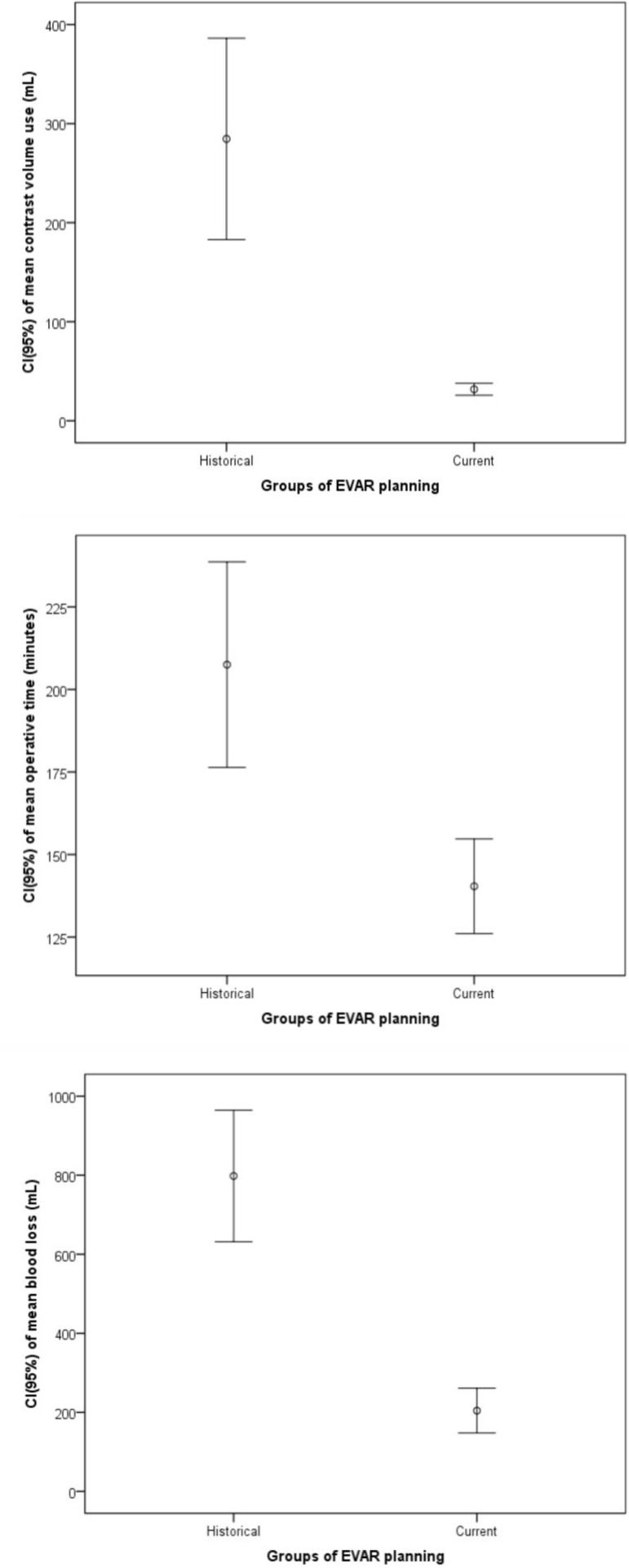
Comparison of surgical variables related to EVAR's preparatory study (Historic *vs.* Script-based current group): contrast volume use (mL), operative time (minutes) and blood loss (mL).

Analyzing the Table 1, it is observed that at the level of significance of 5%, there is a statistically significant relationship between the two periods and the variables: contrast volume (mL), operative time (min.) and blood loss (mL), revealing that they are considerably larger in the historical control group than in the current (Figure 2).

**Table 1:** Group Analysis related to Contrast Volume Use, Total Operative Time and Blood Loss.

Groups of EVAR planning	Historic	Current	Total	*P*-value
**Vol. of Contrast (mL)**				<0.001#
Mean (Stand. Deviation)	284.5 (151.2)	31.8 (16.2)	99.6 (137)	
Median (Minimal - Maximum)	250 (120-550)	28 (10-85)	33 (10 - 55)	
Total	11	30	41	
**Operative Time (minutes)**				<0.001#
Mean (Stand. Deviation)	207.5 (78.7)	140.4 (38.4)	172.2 (69.2)	
Median (Minimal - Maximum)	185 (100 - 415)	130 (85 - 240)	155 (85 - 415)	
Total	27	30	57	
**Blood Loss (mL)**				<0.001#
Mean (Stand. Deviation)	798.1 (420.1)	204.3 (151.6)	485.6 (428.4)	
Median (Minimal - Maximum)	800 (100 - 2000)	150 (50 - 500)	300 (50 - 2000)	
Total	27	30	57	

# t-Student test

In our sample, only one patient in the prospective group and two from the historical group showed a significant increase in serum creatinine levels (i.e., increase in serum creatinine above 25%) compared to baseline. However, there was no statistically significant difference in these findings in relation to the volume of contrast used or preparatory study technique for endovascular repair.

## DISCUSSION

The study of CTA in the preoperative period of EVAR has an essential role in not only planning the type of endoprosthesis that should be used, but also offers the possibility of detailed analysis of the aneurysm morphological characteristics, such as its length, visceral involvement, tortuosity and angulation^[12]^.

Image high-resolution multislice equipment allows refinement of its processing as well as reducing the time of acquisition and improvement of the spatial resolution into thinner slices^[13]^.

Thus, the greater the ability to process these images, the larger is the number of information from the CTA exam can be extracted. Our hypothesis consisted in applying these informations intraoperatively, resulting in less need of contrast use to perform angiograms and thereby reducing surgical time (with consequent lower exposure time to ionizing radiation). This would be achieved by the detailed study of each patient's individual characteristics, with foreknowledge of topographic positioning of visceral arteries and their respective references under fluoroscopic visualization, anticipation of intraluminal positioning of angiographic catheters and correction of the X-ray beam incidence angle of the fluoroscopic device, techniques already published during the pilot study^[10,11]^.

In the present study it was possible to demonstrate the impact of the adoption of a script based on the compilation of these information collected during the preoperative planning, on considerable reduction of intravascular contrast use, surgical time and blood loss, compared to a previous period where this kind of script-based planning did not happen as current.

Consequently, one would expect that the prospective group could present significant outcomes when comparing the volume of contrast used and manifestation of renal toxicity.We did not find, however, that the reduction of the use of contrast was directly related to the reduction in renal overload in both compared groups, because in our sample only one patient in the prospective group and two from historical expressed an increase of 25% of serum creatinine compared to baseline, with no statistical significance. Fortunately, the evolution to acute renal failure is rare^[5]^.

Although is expected a higher incidence of CIN to intraarterial administration compared to intravenous, this known related risk is expected in patients with established chronic kidney disease. For intravenously administered contrast, several studies have shown that the risk of CIN is a glomerular filtration rate (GFR) less than 45 ml/min/1.73m^2^. For intraarterial administration, it maintains a GFR cutoff point of 60 mL/ min/1.73m^2[7,14]^. In our study, 16 patients from the prospective group and 20 from the control group presented such risk, however, into none of these there was a significant increase in serum creatinine in the postoperative period.

There are other risk factors for the occurrence of NIC: diabetic nephropathy, dehydration, congestive heart failure, age over 70, low hematocrit, hypotension and concomitant use of nephrotoxic drugs^[7,14]^. The incidence of CIN is also related to the dose of the used contrast. It is suggested that the dose in grams of iodine is numerically equal to the GFR in mls/min^[7,15]^. While the risk of nephropathy is dose-dependent, it is recommended that patients with less than 60 GFR the administered contrast volume shall be less than 100 ml^[6]^.

Compared to the literature, Alsac et al.^[16]^ cites the average intraoperative use of contrast around 137.5 mL (for infrarenal fixation) and 157.9 mL (for suprarenal fixation stent-grafts). Badger et al.^[17]^ divide between elective and emergency surgery groups, with average contrast use of 163.9 mL for the first and for the second 187.1 mL. Multicentric trials as OVER (Open Versus Endovascular Repair Veterans Affairs Cooperative Study Group, USA)^[18]^ and DREAM (Dutch Randomized Endovascular Aneurysm Management, Holland)^[19]^ describe the mean use of contrast of 132.5 mL e 167 mL, respectively.

Canyigit et al.^[8]^ suggest the categorical selective catheterization of the lowest renal artery using a angiographic catheter Simmons-1 as a proximal landmark during the deployment of the stent graft, reducing the use of contrast to 47 mL per procedure. Regardless of the results, this technique should be considered with reservations since the selective catheterization is not risk free, may be associated with distal embolization, dissection and acute thrombosis. In our study, just through the manipulation and study of CTA images plus adoption of a script-based guide, the average use of contrast decreased to 32 mL - compared to the average historical use of 285 mL. This proves that a meticulous pre-procedure study during EVAR preparation should be incorporated routinely, eliminating unnecessary sequences of image acquisition by digital subtraction.

Despite the CMSC defines that the tracking for diagnostics of CIN takes place in a period of 3 days^[14]^, the short-term TFG can also be influenced by many factors during the perioperative period - such as the duration of the procedure, anesthetic drugs, nephrotoxic contrasts - and so it might not be a reliable evidence of permanent renal dysfunction^[20]^. Therefore, there is the recommendation of postoperative GFR calculation after 1 month of the procedure^[16]^. This conduct is not part of routine in our service and can serve as a suggestion to be adopted for outpatient follow-up.

Prophylactically, it is performed in our Service vigorous hydration before and after surgery with saline solution associated with pre-procedural intravenous administration of NAC. Although some authors advocate that the beneficial effect of this conduct is in the volume expansion with improvement in renal blood flow, in diuresis induction and dilution of the contrast material; others advocate the preferential use of sodium bicarbonate, in suspicion that urine alkalinization decreases the generation of toxic free radicals to the renal parenchyma. Anyway, volumetric expansion (hydration) is considered as degree of recommendation I, evidence level C. The NAC management is controversial, by showing conflicting results in the literature (Class IIb, level of evidence A)^[7]^.

The support of these patients after surgery is performed at the ICU, and hydration is strictly monitored by the attending intensivist. In both groups, for almost all cases, it was observed an improvement in serum creatinine levels compared to baseline, allowing us to assume that the intensive care did not allow the installation of the NIC even in patients at increased risk, while directed to volume expansion and reduction of renal toxicity.

In this type of surgical management, it is justified the patient and medical staff exposure to X-ray, combined with the unique benefits of minimally invasive surgery, as well as minor recovery time and postoperative hospital stay, as an option for patients not elected to conventional open repair^[5]^.

The combined effect over the increasing number of endovascular interventions, complexity and time of procedure associated to the intraoperative coaching to fellows in institutions has become major occupational radiation exposure to the assistant surgeon^[4]^.

This risk-benefit related to the use of radiation should lead doctors to work under minimal exposure required to achieve adequate image quality while allowing safety and effectiveness in procedures. This principle of reduction of X-ray exposure is referred as ALARA (as low as reasonably achieveble). To achieve this goal, different strategies should be combined, since configurations techniques to the X-ray unit up to good medical practice^[5,21]^.

Careful planning that includes CTA image studies with three-dimensional reconstructions allows the surgeon to predict details such as appropriate landmarks of deployment to the different components of the stent-graft. Optimal positioning of the patient and the device often can be established prior to the procedure, then fully reducing the total fluoroscopic surgical time^[3]^.

Under virtual fluoroscopy, the data manipulation of the irradiated dose distribution on a surface allows to visualize as opaque areas of high contrast (like bone surfaces) and as transparent low attenuation values (soft tissue). Guided by marks of the renal arteries, it can be carefully foreseen its anatomical position in relation to its visualization on real-time fluoroscopy. The anticipation of the correct positioning of the radioscopic unit with this technique use allows obtaining image with the minimum of interference from the parallax effect. It is therefore possible decrease the number of angiograms in trying to obtain the best image that provides the localization of renal arteries and the aneurysm neck^[11]^.

The intraoperative image during EVAR is limited to two-dimensional details that depict only the arterial lumen and contrasting requires repeated acquisitions. To accomplish optimal outcomes the surgeon must comprehend the geometry of the fluoroscopic image formation and the various adjustments to the C-arm unit that can clarify more complex vascular anatomy. When angulating the X-ray tube in order to fix its incidence for a perpendicular beam to the axis of the aneurysm neck (proximal margin of sealing), the endoprosthesis presents itself in its ideal position with no risk of accidental coverage of the renal artery^[3]^. This effect can be achieved by the renal artery's ostial projection under virtual geometric correction, implemented through OsiriX software^[10]^.

As a result of the adoption of this new protocol in the present analysis, there was found a significant decrease of surgical time. Therefore, one can assume a substantial reduction in the exposure to ionizing radiation to patients as well as a lower exposure of the interventionalist team. But when compared to literature data, this average fluoroscopy time was longer. For the same kind of procedure, also performed with a C-arm, Geijer, Weiss, and Maurel Kalef-Ezra reported respectively mean values of 28.4, 20.6, 22.6 and 11.2 min. (*vs.* 33.3 min.)^[5]^. This further reinforces a constant need for specific protective training for radiation exposure, use of barrier vests, beam collimators and enhancement in efforts to cut down on unnecessary fluoroscopy, when the surgeon is not directly being guided by the X-ray image in exchange of catheters or saline infusion.

Bicknell^[22]^ emphasizes that simple measures to protect the surgical team to the exposure may not be enough. Thus, technological advances in low-dose imaging presented by large companies in this field are a major step in reducing radiation dose while maintaining image quality for performing complex procedures.

This process is vital in continuous techniques that reduce the need for repeated DSA. The fusion of either pre- or intraoperative sectional images is clearly effective in reducing the radiation exposure - and other imaging technologies such as intravascular ultrasound can complement these approaches. Further, the use of advanced technology will allow the surgeon's withdrawal from source of radiation, as in robotic surgery (Magellan, Hansen Medical, Mountain View, CA, USA)^[22]^. Nevertheless, it is noteworthy that the cost of these new technologies makes them a limited access, not allowing its wide spread use, only for major international technology centers.

Although it's known that EVAR is associated with less blood loss when compared to the conventional approach by open surgery, the bleeding can also occur through the puncture sites and around large introducer sheaths - by handling of catheters and guidewires as well as complications during the closure of the access sites^[23]^. Thus, unnecessary maneuvering, repeated exchanges of catheters of different formats, sheaths and rigid guide wires (which in turn may force the introducer's diaphragm leading to malfunction of hemostatic valves) significantly increase the bleeding during the procedure. In the present study, there was a significant reduction to about a quarter the bleeding in the prospective arm compared to the historical control group (204.3 *vs.* 798.1 mL), while loss are described in the literature from 200 mL to 394 mL^[18,19]^. Thus, it is realized that the preparation and anticipation of the steps promoted by the script allow surgical technique with perfection and eliminating additional steps that would have little impact on the course of surgery and it certainly would collaborate to a massive bleeding.

The main limitation of this study was to monitor the variables for the retrospective nature of the historical control group because it was not possible to compare to the prospective arm data of irradiation dose as well as fluoroscopy time and endovascular execution, not described in the medical record - plus the occurrence of replacement of the X-ray equipment by another one of distinct brand in this interval. This is also a fellowship scenario where the trainees participate actively in the surgical procedure and have their own learning curve - that may have influenced total operating time and fluoroscopy.

## CONCLUSION

This study presents a simple technique and of great practical importance in planning interventional treatments. The ability to manipulate digital formats of medical images allows the recovery of a larger volume of data and allows that interventional procedures can be performed more efficiently, with less time for image projection adjustment, contrast injections and exposure to ionizing radiation. As a result, we obtained impact in relation to the improvement of the surgical technique, translated into less use of contrast, reduced surgical time and intraoperative bleeding.

**Table:** 

**Authors' roles & responsibilities**	
GJDPM	Analysis and/or interpretation of data; statistical analysis; design and drawing of the study; implementation of projects and/or experiments; manuscript writing or critical review of its contents; final approval of the manuscript
ATG	Execution of operations and/or experiments; manuscript writing or critical review of its contents; final approval of the manuscript
AMOD	Realization of operations and/or experiments; manuscript writing or critical review of its contents; final approval of the manuscript
